# Is weight gain preventable in women with early breast cancer undergoing chemotherapy? A real-world study on dietary pattern, physical activity, and body weight before and after chemotherapy

**DOI:** 10.1007/s10549-023-07095-8

**Published:** 2023-09-11

**Authors:** Rebecca Pedersini, Marta Laganà, Sara Bosio, Barbara Zanini, Deborah Cosentini, Pierluigi di Mauro, Andrea Alberti, Greta Schivardi, Lara Laini, Giuseppe Ippolito, Vito Amoroso, Lucia Vassalli, Edda Lucia Simoncini, Alfredo Berruti, Francesco Donato

**Affiliations:** 1 Medical Oncology Unit, Department of Medical and Surgical Specialties, Radiological Sciences and Public Health, University of Brescia, ASST Spedali Civili of Brescia, Piazzale Spedali Civili 1, 25123 Brescia, Italy; 2 SSVD Breast Unit, ASST Spedali Civili of Brescia, 25123 Brescia, Italy; 3 Clinical and Experimental Sciences Department, University of Brescia, 25123 Brescia, Italy; 4 Unit of Hygiene, Epidemiology, and Public Health, Department of Medical and Surgical Specialties Radiological Sciences and Public Health, University of Brescia, 25123 Brescia, Italy

**Keywords:** Breast cancer, Adjuvant chemotherapy, Dietary habits, Physical activity, Weight change, BMI

## Abstract

**Purpose:**

We aimed to investigate the role of a lifestyle intervention and clinical and therapeutic factors for preventing weight gain in early breast cancer (BC) patients from one week before to 12 months after chemotherapy.

**Methods:**

Dietary assessments were conducted by a trained dietician using a food-frequency questionnaire at each clinical assessment. Total energy, macronutrients intakes, and physical activity were estimated and the Mediterranean Diet Score (MDS) for adherence to Mediterranean diet was calculated. At each follow-up visit, patients were provided with dietary advices according to Mediterranean and Italian guidelines by a registered dietician, after evaluation of their food records. The associations of clinical characteristics, dietary pattern, and physical activity with weight gain were evaluated by multiple logistic regression, with weight gain ≥5% from baseline value as a dichotomous dependent variable.

**Results:**

169 early BC patients who met all follow-up visits and provided complete data were included in the analysis. From baseline to last assessment, weight loss (≥5% decrease from baseline value), stable weight, and weight gain were observed in 23.1%, 58%, and 18.9% women, respectively. Overall, a 0.68 kg mean decrease in women’s weight (−1.1% from baseline) was observed. The risk of gaining weight increased for having normal weight/underweight at baseline, receiving hormone therapy, MDS worsening, and physical activity decreasing from baseline to last assessment.

**Conclusion:**

Providing simple suggestions on Mediterranean diet principles was effective for preventing weight gain in normal weight women and favoring weight loss in overweight and obese women.

**Supplementary Information:**

The online version contains supplementary material available at 10.1007/s10549-023-07095-8.

## Introduction

Several breast cancer (BC) survivors have significant weight gain after diagnosis [1], and strategies for preventing obesity are included in the American Cancer Society/American Society of Clinical Oncology Breast Cancer Survivorship Care Guideline [2, 3]. Indeed, weight gain and obesity after breast cancer diagnosis have been found to increase the risk of BC recurrence and mortality, and of all-cause mortality [4–7]. Adjuvant therapy often includes endocrine treatment because 60–80% of early BC tumors are hormone receptor (HR)-positive, which has been associated with weight gain in some but not all studies [8]. To the best of our knowledge, no previous studies have evaluated the role of adjuvant endocrine therapy in gaining weight when also considering dietary habits and physical activity, which are supposed to be major determinants of weight gain.

Preventing weight gain or promoting weight loss in BC patients undergoing chemotherapy was achieved in some but not all randomized controlled trials (RCTs) based on dietary and physical activity interventions [9]. A meta-analysis of RCTs and a systematic review of reviews found that dietary-based interventions, with or without physical activity improvement, resulted in a significant decrease of weight in women with BC [10, 11]. The Mediterranean diet (MD) is considered a healthy dietary pattern, and the adherence to MD has been found to reduce the risk of chronic disease occurrence [12, 13] and the risks of all-cause mortality in BC survivors [14] and it is effective for reducing body weight in BC patients in dietary interventions [15]. However, the dietary and physical activity interventions carried out in effective RCTs require a considerable amount of resources and are, therefore, difficult to be included in routine activity in Breast Cancer Units. In a previous study, we showed that the simple collection of BC patients’ dietary habits by a dietician was effective in preventing weight gain from before to immediately after chemotherapy [16]. After the end of chemotherapy, however, the dietician also provided suggestions on healthy diet, according to the principles of the Mediterranean diet and in line with the Italian Society of Human Nutrition guidelines [17, 18], at each subsequent 3-month follow-up visit. Therefore, we extended our observation to 12 months after the completion of chemotherapy.

The primary aim of this study was to investigate the effect of a simple lifestyle intervention, as well as that of clinical and therapeutic factors, in preventing weight gain in early BC patients up to 12 months after the end of chemotherapy. A secondary aim of the study was to evaluate the associations of the same factors with weight loss in overweight and obese patients.

## Patients and methods

We conducted a single-center intervention study at the Medical Oncology and Breast Unit of the ASST Spedali Civili of Brescia, Italy, after registration in the ClinicalTrials.gov database (NCT identification number: NCT03210441) and approval by the local Ethics Committee of ASST Spedali Civili of Brescia. From April 2014 to June 2018, we consecutively approached all women referred to Medical Oncology and Breast Unit to plan adjuvant or neo-adjuvant chemotherapy for early BC. Eligibility criteria included histologically confirmed BC, indication for adjuvant or neo-adjuvant chemotherapy, regardless of tumor biology and menopausal status, and understanding of the Italian language. We obtained written informed consent from all participants.

The clinical data were retrieved from the Breast Unit records. The dietary assessment was conducted by a trained dietician using a questionnaire specifically developed for this study according to the methodology previously described [16], with the following timeline: 1 week before starting chemotherapy (baseline, T0), 2 months after starting chemotherapy (T1), 1 week after the end of chemotherapy (T2), and at follow-up 3-month visits up to 12 months after the end of chemotherapy (T3, T4, T5, and T6). The Mediterranean Diet Score (MDS) has been proposed for measuring the degree of adherence to the traditional Mediterranean diet [19]. At each visit, BC patients’ weight and height were measured and data on employment, physical activity, and alcohol consumption were collected. BMI was calculated as the ratio of kgs of body weight to the square of height in meter. Physical activity was defined as 150 min and over per week of moderate or intense physical exercise. Data on physical activity frequency, intensity, and type were also collected. The dieticians who collected all the patients’ data provided general advice on healthy diet according to the latest version of food-based Italian dietary guidelines, published by CREA [18]. These advices were provided only after the end of chemotherapy, during subsequent follow-up visits (T3–T6).

### Statistical methods

The sample size calculation for the CHANGE study has been detailed in a previous paper (Pedersini 2021). Briefly, the sample size was calculated assuming weight gain from before to after chemotherapy in 50% of patients, with a maximum difference of ±10%. Considering a type I error of 5% and a power of 85%, the number of patients to be included in the study was 177, and, assuming 5% of dropouts, we established to enroll 204 BC patients. Categorical and continuous variables were reported as frequencies and percentages, and means and relative 95% confidence intervals (CIs), respectively. For evaluating the changes in the investigated parameters during the study period, we made comparisons between the data collected one week before chemotherapy and 12 months after the end of chemotherapy, for a total of 18 months of interval time. For continuous variables, since almost all of them showed skewed, non-normal distributions, we used non-parametric tests for paired comparisons, particularly the Wilcoxon signed-rank test. For categorical variables, we used the Chi square test, and the Fisher’s exact test when the assumptions for the former were not met.

The weight change was calculated as the difference between the measures taken 12 months after chemotherapy and 1 week before chemotherapy (baseline), divided by the baseline weight to provide a percentage change. An absolute value of weight gain or loss of 5% or more was considered clinically meaningful, in line with others [20]. Accordingly, weight difference from baseline and last assessment was considered a weight gain or loss for an increase or a decrease of at least 5% from baseline, respectively, and stable weight otherwise. The associations between the clinical characteristics, dietary pattern, physical activity, and weight gain were evaluated by univariate analysis using the common statistical tests for the analysis of continuous and categorical variables. Multivariable analysis was also performed using logistic regression models with weight gain (yes/no) as a dichotomous dependent variable. The selection of variables included in the final, parsimonious model was made by backward procedure, using a cut-off of *p* = 0.05 for the Wald test on coefficients for retaining each variable in the regression model. The results were expressed as odds ratios (ORs) and their 95% confidence intervals (95% CIs). All the statistical tests were two-sided with 5% threshold for rejecting the null hypothesis. All the analyses were performed using the Stata software for pc, vs. 17 (Stata Corp, LP).

## Results

A total of 215 early BC patients were enrolled and underwent the baseline dietary and physical activity assessment one week before starting chemotherapy (baseline, T0). Among them, 169 patients met all the following visits up to the last assessment, 12 months after the end of chemotherapy (from T1 to T6). The remaining 46 women abandoned the study during the follow-up and did not attend the final visit (T6), and therefore, were excluded from the analysis.

The physiological and clinical characteristics of the BC patients at baseline are shown in Table 1. The mean age was 54 years (range 25–80 years). The majority of them had a normal weight or underweight, were postmenopausal, had no lymph node involvement, high grade tumors and were positive for estrogen and progesterone receptors. The sequential regimen with anthracyclines and taxanes was the most used treatment, and the majority received also hormone therapy. All premenopausal patients reported chemotherapy-induced amenorrhea at the end of the adjuvant treatment. The baseline physiological and clinical characteristics of the 46 BC patients who did not undergo the last assessment are shown in Table 1 of the supplementary material. No statistically significant difference was found in physiological and clinical characteristics between the compliant (see Table 1) and the non-compliant BC patients for each variable.

**Table 1:** Clinical characteristics at baseline (1 week before starting chemotherapy) (*n* = 169)

Characteristics	*N* (%)
Age, years: mean ± SD (range)	54 ± 11 (25;80)
BMI	
Normal weight/underweight	102 (60.4)
Overweight	41 (24.3)
Obesity	26 (15.4)
Mean ± SD (range)	24.9 ± 4.8 (15.9;41.2)
Menopausal status	
Premenopausal	68 (40)
Postmenopausal	101 (60)
Pathological tumor stage	
0	19 (11)
1	82 (49)
≥2	68 (40)
Pathological nodal stage	
0	95 (56)
≥1	74 (44)
Histological type	
No special type	153 (91)
Others	16 (9)
Estrogen receptor	
Positive	118 (70)
Negative	51 (30)
Progesterone receptor	
Positive	99 (59)
Negative	70 (41)
Grading	
G1 or G2	20 (12)
G3	147 (87)
Unknown	2 (1)
Ki-67 labeling index	
<20%	31 (18)
≥20%	138 (82)
HER2	
Positive	67 (40)
Negative	102 (60)
Chemotherapy	
Adjuvant	130 (78)
Neo-adjuvant	39 (22)
Chemotherapy regimen	
Anthracyclines	29 (17)
Taxanes	22 (13)
Sequential	109 (65)
Others	9 (5)
Hormone therapy	
None	56 (33)
Tamoxifen	13 (8)
Aromatase inhibitors	83 (49)
LHRH agonist + tamoxifen	6 (4)
LHRH agonist + aromatase inhibitors	11 (6)
Surgery treatment	
Breast-conserving	91 (54)
Mastectomy	78 (46)

*G1* well-differentiated tumor, *G2* moderately differentiated tumor, *G3* undifferentiated tumor, *HER2* human epidermal growth factor receptor 2, *LHRH* luteinizing hormone-releasing hormone

The mean consumption of foods, beverages, and condiments at baseline and last assessment, 12 months after the end of chemotherapy (T6), in grams or milliliters per week, is reported in Table 2. A statistically significant change was observed in the mean week intake of most foods and beverages, with an increased intake of fruit, vegetables, fish, and legumes and a decreased intake of pasta or rice, bread, potatoes, breadsticks/crackers, white and red meat, lean and fat salami, aged cheese, milk and yogurt, added sugar, icecreams and sweet snacks, soft drinks and fruit juices and alcoholic beverages, particularly wine, and butter. No significant changes were observed for the other items. The proportion of women reporting a daily alcohol consumption declined from 59.8% to 48.5%.

**Table 2:** Food, beverages, and condiments consumption 1 week before starting chemotherapy (T0) and 12 months after the end of chemotherapy (T6)

Food, beverage or condiment	T0	T6	ΔT6–T0	p*
Fruit	1852 (1669;2035)	2179 (2032;2327)	+328 (125;530)	0.002
Vegetables	1579 (1463;1964)	1868 (1786;1950)	+290 (172;407)	<0.0001
Pasta or rice	393 (362;425)	343 (316;370)	−49 (−83;−16)	0.002
Bread	429 (382;476)	296 (262;331)	−133 (−184;−82)	<0.0001
Potatoes	271 (236;305)	209 (183;235)	−62 (−102;−22)	0.008
Breadsticks or crackers	103 (83;124)	30 (20;41)	−73 (−93;−52)	<0.0001
White meat	242 (217;267)	207 (189;224)	−35 (−60;−10)	0.008
Red meat	135 (117;153)	76 (66;87)	−59 (−78;−40)	<0.0001
Fish	271 (241;301)	361 (331;392)	+90 (58;121)	<0.0001
Lean salami	99 (85;114)	36 (28;44)	−63 (−78;−48)	<0.0001
Fat salami	29 (22;36)	6 (4;9)	−23 (−29;−16)	0.052
Eggs	118 (105;131)	116 (105;127)	−2 (−17;13)	0.81
Fresh cheese	200 (172;228)	170 (150;191)	−30 (−62;3)	0.31
Aged cheese	124 (106;142)	60 (51;70)	−63 (−82;−44)	<0.0001
Legumes	205 (173;237)	321 (289;353)	+116 (78;154)	<0.0001
Milk	449 (353;544)	232 (161;303)	−216 (−316;−117)	<0.0001
Yogurt	336 (249;423)	110 (72;149)	−226 (−318;−133)	<0.0001
Added sugar	75 (61;89)	25 (17;33)	−50 (−63;−37)	<0.0001
Biscuits	135 (115;15)	112 (94;130)	−23 (−45;1)	0.11
Icecreams	85 (64;106)	32 (19;46)	−53 (−76;−30)	<0.0001
Sweet snacks	176 (137;217)	77 (58;96)	−99 (−138;−59)	<0.0001
Soft drinks	259 (172;347)	57 (16;98)	−202 (−279;−126)	<0.0001
Fruit juices	246 (182;311)	89 (49;129)	−158 (−226;−90)	<0.0001
Wine	305 (228;381)	190 (133;246)	−115 (−181;−49)	0.007
Beer	122 (78;165)	64 (43;85)	−58 (−102;−13)	0.11
Spirits	1.8 (0.1;3.4)	1.4 (0.2;3.1)	−0.4 (−2.7;2.0)	0.50
Olive oil	249 (236;261)	245 (235;256)	−3 (−19;13)	0.58
Butter	19 (14;23)	9 (6;12)	−10 (−15;−5)	<0.0001

All measures are expressed as means of grams for food and ml for beverages per week, with the corresponding 95% confidence interval in brackets

*Wilcoxon signed-rank test

**Fig. 1:**
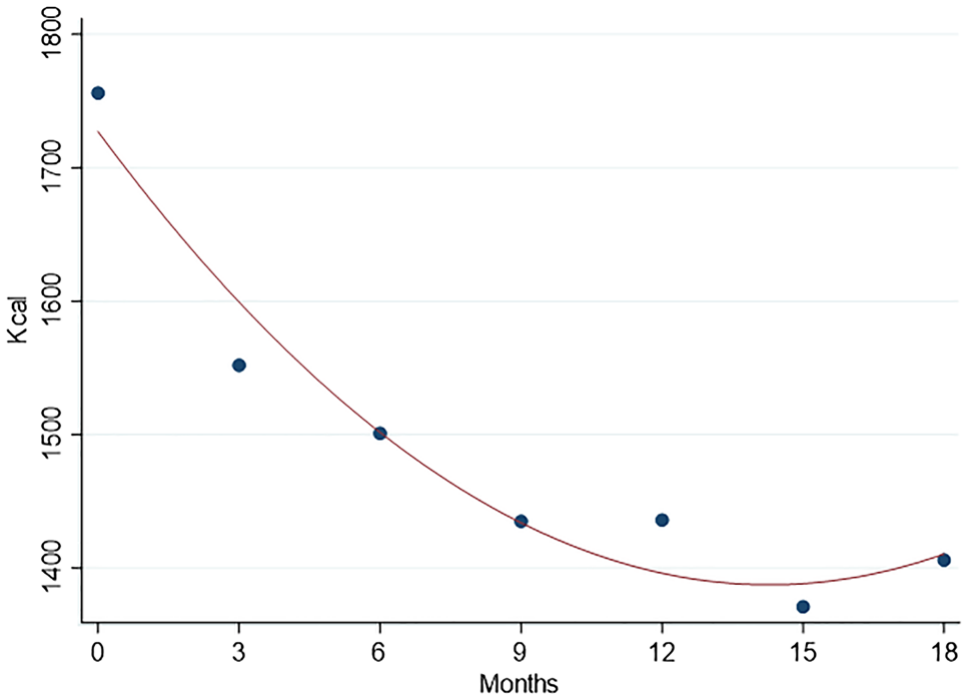
Time trend of the mean daily kcal intake, from baseline (1 week before start of chemotherapy) to 18 months (12 months after the end of chemotherapy). The line was fitted including a quadratic term in the regression model

**Fig. 2:**
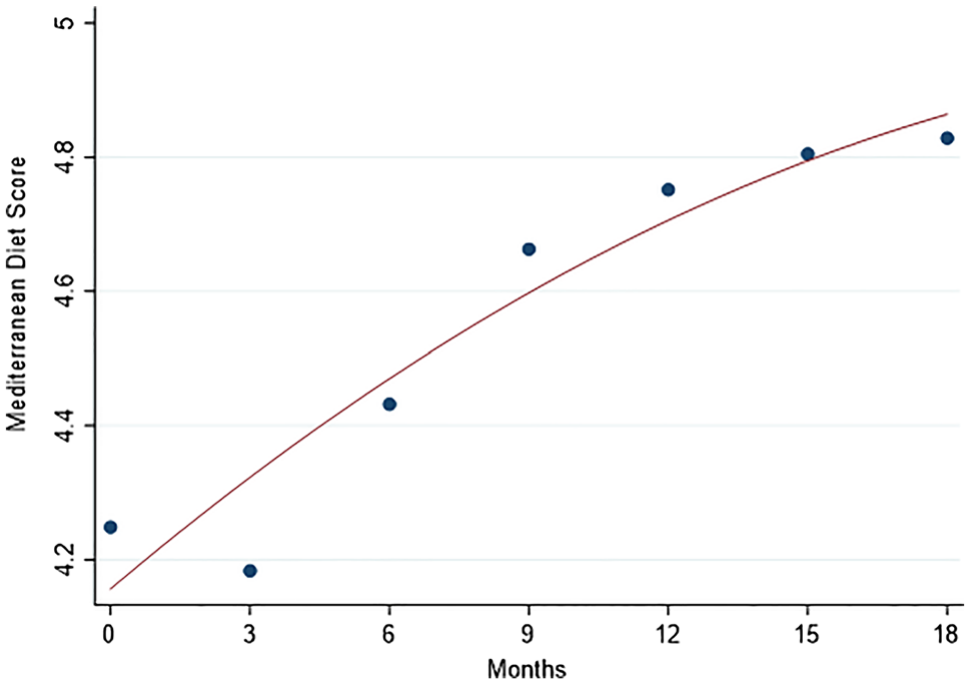
Time trend of the mean Mediterranean Diet Score (MDS) from baseline (1 week before start of chemotherapy) to 18 months (12 months after the end of chemotherapy). The line was fitted including a quadratic term in the regression model

The baseline consumption of foods, beverages, and condiments in the BC patients who did not undergo the last assessment is shown in Table 2 of supplementary material. A comparison with the BC patients who participated in the whole study (see Table 2) did not show significant differences for each variable. A significant decrease in daily calorie intake, of about 350 kcal (95% CI: from 277 to 424 kcal), was observed from baseline to last assessment, due to a reduction of macronutrients intake (proteins, carbohydrates, and fat), as shown in Table 3. An increase in fiber intake was also seen. The mean MDS improved of 0.6 in a 9 points scale, in agreement with the changes shown in single foods and beverages intake (see Table 2). The time trend of the mean kcal daily intake, from baseline to 12 months after the end of chemotherapy (18 months of observation time) is reported in Fig. 1. Fitting a regression model with a quadratic term, a deep decline of kcal daily intake was apparent from baseline to 9th month (about 3 months after ending chemotherapy), with a flattening of the curve thereafter. A significant increase in the MDS was observed in the same time, with no clear trend at the end of the period (Fig. 2). The proportion of women who practiced physical activity increased from baseline (49.1%) to last assessment (73.4%), with an increase of the proportion who reported physical activity for 3–7 days a week, from 33.7% to 49.1% (data not shown in tables). Most women had an employment at both baseline and last assessment (70.4% and 61.5%, respectively).

**Table 3:** Calorie intake, macronutrients, and Mediterranean Diet Score 1 week before starting chemotherapy (T0), and 12 months after the end of chemotherapy (T6)

Daily intake	T0	T6	Δ T6-T0	p*
Total energy (kcal)	1751 (1683;1830)	1406 (1363;1449)	−350 (−424;−277)	<0.0001
Proteins (g)	59.1 (56.7;61.5)	48.9 (47.2;50.6)	−10.2 (−12.7;−7.7)	<0.0001
Proteins (kcal)	237 (227;246)	196 (189;202)	−41 (−51;−31)	<0.0001
Proteins energy percentage (%)	13.7 (13.3;14.0)	14.0 (13.7;14.4)	+0.3 (−0.1–0.8)	0.049
Carbohydrates (g)	204 (194;215)	167 (160;174)	−37 (−48;−27)	<0.0001
Carbohydrates (kcal)	817 (776:859)	669 (640;698)	−149 (−191;−106)	<0.0001
CHO energy percentage (%)	46.2 (45.1;47.4)	47.3 (46.3–48.3)	+1.0 (−0.3;2.4)	0.12
Sugars energy percentage (%)	15.1 (14.3;16.0)	14.4 (13.7;15.1)	−0.7 (−1.7;0.2)	0.09
Fibers (g)	19.8 (18.7;20.8)	22.3 (21.6;23.1)	+2.6 (1.5;3.7)	<0.0001
Fats (g)	71.9 (68.6;75.3)	58.3 (56.4;60.2)	−13.7 (−17.2;10.1)	<0.0001
Fats (kcal)	648 (617;678)	525 (508;541)	−123 (−155;−91)	<0.0001
Fast energy percentage (%)	37.0 (36.0;38.0)	37.7 (36.7;38.6)	+0.6 (−0.6;1.9)	0.44
Saturated fats energy percentage (%)	11.2 (10.8;11.6)	11.1 (10.7;11.5)	−0.1 (−0.5;0.3)	0.89
MDS (score 0–9)	4.2 (4.0;4.5)	4.8 (4.6;5.1)	+0.6 (0.3–0.8)	<0.0001

All measures are expressed as means of grams for food and ml for beverages per day, with the corresponding 95% confidence interval in brackets

*kcal* kilocalories, *g* grams, *MDS* Mediterranean Diet Score [19]

*Wilcoxon signed-rank test

**Fig. 3:**
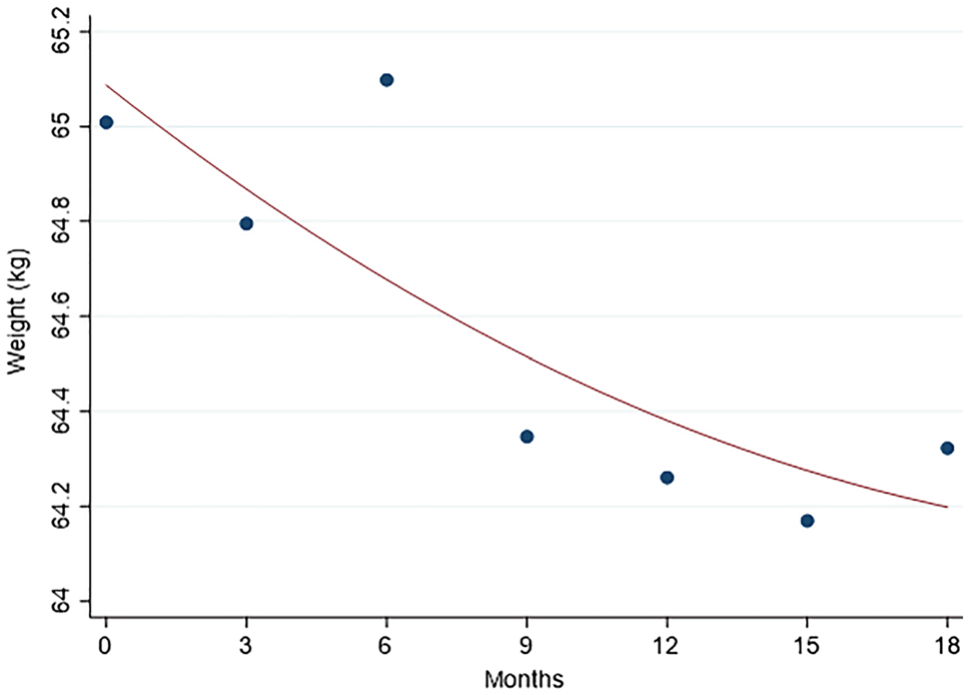
Time trend of the mean weight from baseline (1 week before starting chemotherapy) to 18 months (12 months after the end of chemotherapy). The line was fitted including a quadratic term in the regression model

From baseline to last assessment, weight loss (≥5% the baseline value), stable weight and weight gain (≥5% baseline value) were observed in 23.1%, 58%, and 18.9% women, respectively. Overall, a 0.68 kg mean decrease in women’ s weight (−1.1% of baseline) was observed (95% CI: −0.07; −1.30; *p* = 0.05 by Wilcoxon test), with a wide variability, from −13 kg to +10.5 kg. The decline of the mean weight was linear from the end of chemotherapy, 6th month after baseline, to 15th month, and remained stable thereafter (Fig. 3). The weight gain was more frequent among younger than older women, and among those with normal weight/underweight than overweight and obese women (Table 4). About one third of overweight and obese women lost weight in the study period (39.0% and 30.8%, respectively), whereas very few of them gained weight (4.9% and 3.9%, respectively). No clinical characteristic was associated with weight change, apart from presence of progesterone receptors: 25.3% and 10% of women had weight gain among those positive and negative for progesterone receptors, respectively. The proportion with weight gain was also higher in women who received hormone therapy than in those who did not (23% and 10.7%, respectively). No association was found between weight change and calorie intake, whereas weight gain was observed in a higher proportion of women with MDS worsening (44.4%) than in those with no MDS change or improvement (14.4% and 18.5%, respectively). Similarly, a higher proportion of subjects with weight gain was found among women who reduced physical activity (38.5%) than those who maintained or increased physical activity (16.7% and 14.1%, respectively). As regard to hormone therapy, no difference was found between 113 women receiving and 56 not receiving hormone therapy, for foods, beverages and condiments mean consumption, total energy intake, and MDS, at both baseline and last assessment, as shown in Table 3 of the supplementary material. Women who received hormone therapy and those who did not receive it had similar mean calorie intakes, from baseline to last assessment: they reduced calorie intake of 304 and 442 kcal/day (difference: −137, 95% CI: −293, 17), increased MDS of 0.5 and 0.8 points (difference: −0.3, 95% CI: −0.9, 0.2) and lost 0.3 and 1.4 kg of weight (difference: −1.1, 95% CI: −2.4, 0.2), on the average, respectively. According to BMI categories, some women had an improvement and few a worsening, from baseline to last assessment: 9/102 (8.8%) of those with normal weight or underweight at baseline passed to overweight, and 2/41 (4.9%) women with overweight at baseline advanced to obesity. On the contrary, 12/41 (29.3%) overweight women at baseline passed to normal weight and 8/26 (30.8%) obese women at baseline passed to overweight. Finally, the associations between physiological and clinical characteristics and dietary and physical activity habits and weight gain were assessed using univariate and multivariable methods. To this end, body weight gain was dichotomized as yes/no, collapsing the categories of “stable weight” and “weight loss” into the “no gain” category. The proportion of women gaining weight was significantly higher among those aged less than 59 years, with normal weight at baseline, with progesterone receptors, who received hormone treatment, with worsening MDS and with reducing physical activity (Table 4, supplementary material). The results of multivariable analysis of the associations between weight gain and investigated variables using logistic regression analysis, with weight gain as dichotomous dependent variable, and the odds ratios (ORs) and their 95% CIs as measures of associations, are shown in Table 5. The risk of gaining weight (≥5% baseline) decreased with increasing age, and increased in women who received hormone therapy, had a normal weight/underweight at baseline, had MDS worsening and a physical activity decrease from baseline to last assessment. The OR estimates were particularly high but imprecise, as shown by large CIs, due to the relatively small number of women in some categories. Type of chemotherapy was not associated with weight gain and no differences in the OR estimates were observed when the analysis was restricted to women treated with aromatase inhibitors (data not shown in Tables). When restricting the analysis to BC patients with hormone therapy, similar results were observed, though the OR estimates were less preciese due to the smaller number of observations (*n* = 113): the ORs for weight gain for age, normal weight at baseline, MDS worsening, and physical activity decrease were 0.96 (95% CI: 0.0.91, 1.01), 8.63 (2.09, 35.65), 7.58 (1.78, 32.31), and 3.62 (1.07, 12.26), respectively (data not shown in tables).

**Table 4:** Characteristics of the patients, dietary kcal intake, Mediterranean Diet Score (MDS), and physical activity frequency according to body weight change from the start of chemotherapy to 12 months after the end of chemotherapy

Characteristics	Weight loss	Stable weight	Weight gain	Total	P*
	*N* (%)	*N* (%)	*N* (%)	*N* (%)	
Total patients	39 (23.1)	98 (58)	32 (18.9)	169 (100)	
Age					0.033
<49	8 (15.69)	26 (50.98)	17 (33.33)	51 (100)	
49–58	16 (27.12)	35 (59.32)	8 (13.56)	59 (100)	
>59	15 (25.42)	37 (62.71)	7 (11.86)	59 (100)	
Basal BMI					<0.001
Normal weight/underweight	15 (14.71)	58 (56.86)	29 (28.43)	102 (100)	
Overweight	16 (39.02)	23 (56.10)	2 (4.88)	41 (100)	
Obesity	8 (30.77)	17 (65.38)	1 (3.85)	26 (100)	
Menopausal status					0.456
No	15 (22.06)	37 (54.41)	16 (23.53)	68 (100)	
Yes	24 (23.76)	61 (60.40)	16 (15.84)	101 (100)	
Pathological tumor stage					0.944
0	4 (21.05)	11 (57.89)	4 (21.05)	19 (100)	
1	18 (21.95)	50 (60.98)	14 (17.07)	82 (100)	
≥ 2	17 (25)	37 (54.41)	14 (20.59)	68 (100)	
Pathological nodal stage					0.467
0	20 (21.05)	59 (62.11)	16 (16.84)	95 (100)	
≥1	19 (25.68)	39 (52.70)	16 (21.62)	74 (100)	
Histologic type					0.935
No special type	36 (23.53)	88 (57.52)	29 (18.95)	153 (100)	
Lobular	3 (20)	9 (60)	3 (20)	15 (100)	
Others	0 (0)	1 (100)	0 (0)	1 (100)	
Estrogen receptor					0.430
Negative	11 (21.57)	33 (64.71)	7 (13.73)	51 (100)	
Positive	28 (23.73)	65 (55.08)	25 (21.19)	118 (100)	
Progesterone receptor					
Negative	17 (24.29)	46 (65.71)	7 (10)	70 (100)	0.042
Positive	22 (22.22)	52 (52.53)	25 (25.25)	99 (100)	
Grading					0.484
G1 or G2	6 (30)	9 (45)	5 (25)	20 (100)	
G3	33 (22.45)	87 (59.18)	27 (18.37)	147 (100)	
Ki-67 labeling index					0.544
<20%	7 (22.58)	16 (51.61)	8 (25.81)	31 (100)	
≥20%	32 (23.19)	82 (59.42)	24 (17.39)	138 (100)	
HER2					0.328
Negative	23 (22.55)	56 (54.90)	23 (22.55)	102 (100)	
Positive	16 (23.88)	42 (62.69)	9 (13.43)	67 (100)	
Chemotherapy					0.908
Adjuvant	29 (22.31)	76 (58.46)	25 (19.23)	130 (100)	
Neo-adjuvant	10 (25.64)	22 (56.41)	7 (17.95)	39 (100)	
Chemotherapy regimen					0.659
Taxane based	30 (22.90)	78 (59.54)	23 (17.56)	131 (100)	
Nontaxane based	9 (23.68)	20 (52.63)	9 (23.68)	38 (100)	
Hormone therapy					0.151
No	15 (26.79)	35 (62.50)	6 (10.71)	56 (100)	
Yes	24 (21.24)	63 (55.75)	26 (23.01)	113 (100)	
Type of surgery					0.673
Conservative	22 (24.18)	54 (59.34)	15 (16.48)	91 (100)	
Mastectomy	17 (21,79)	44 (56.41)	17 (21.79)	78 (100)	
Kcal T0–T6					0.43
Mean difference ± SD	−433 ± 430	−309 ± 483	−372 ± 543	−350 ± 483	
Decrease	28 (25.45)	60 (54.25)	22 (20)	110 (100)	
Stable	9 (25)	21 (58.33)	6 (16.67)	36 (100)	
Increase	2 (8.70)	17 (73.91)	4 (17.39)	23 (100)	
MDS T0–T6					0.014
Mean difference ± SD	0.84 ± 1.58	0.54 ± 1.64	0.38 ± 2.02	0.58 ± 1.71	
Worsening	3 (16.67)	7 (38.89)	8 (44.44)	18 (100)	
Stable	19 (19.59)	64 (65.98)	14 (14.43)	97 (100)	
Improvement	17 (31.48)	27 (50)	10 (18.52)	54 (100)	
Physical activity frequency T0–T6					
Decrease	4 (15.38)	12 (46.15)	10 (38.46)	26 (100)	0.094
Stable	18 (25)	42 (58.33)	12 (16.67)	72 (100)	
Increase	17 (23.94)	44 (61.97)	10 (14.08)	71 (100)	

*MDS* Mediterranean Diet Score [19]

*Chi square o exact test. Weight loss and weight gain from the 1 week before starting chemotherapy (baseline) to 12 months after the end of chemotherapy: ≥5% of baseline body weight.

**Table 5:** Characteristics associated with weight gain (≥5% baseline value), from baseline to 12 months after chemotherapy, fitting a logistic regression model: odds ratios (ORs) and corresponding 95% confidence intervals (CIs)

Characteristic	No. (%) with weight gain/total	OR	95% CI	*p*
Age (years)		0.94	0.90–0.98	0.015
Hormone therapy				
No	6/56 (10.7)	Reference		
Yes	26/113 (23.0)	3.26	1.06–10	0.04
Baseline weight				
Overweight/obesity	3/67 (4.5%)	Reference		
Normal weight/underweight	29/102 (28.4)	10.56	2.59–43	0.001
MDS T0–T6				
Stable/improvement	24/151 (15.9)	Reference		
Worsening	8/18 (44.4)	7.08	1.94–25	0.003
Physical activity frequency T0–T6				
Stable/increase	22/143 (15.4)	Reference		
Decrease	10/26 (38.5)	4.90	1.59–15	0.006

*MDS* Mediterranean Diet Score [19]

## Discussion

The main finding of this real-life study is that the risk of gaining weight in BC patients treated with chemotherapy decreased with increasing adherence to Mediterranean diet (MD) and with maintaining or increasing physical activity, and increased with hormone treatment. Furthermore, this study shows that providing some simple suggestions to BC patients for healthy diet and practice of physical exercise at each follow-up visit may prevent weight gain, and favor weight loss in those who are overweight or obese. BC patients lost a mean of 0.68 kg of body weight, from one week before to 12 months after ending chemotherapy, with a linear trend in the study period. Most patients maintained their weight (58%), and the proportion of those with ≥5% weight loss (23.1%) was moderately higher than that of patients with ≥5% weight gain (18.9%). Women with normal weight at baseline were at higher risk of gaining weight than overweight or obese women, in agreement with other studies [21, 22], probably because overweight and obese women were more motivated to lose weight for ameliorating the prognosis of their disease. In contrast with several studies that found weight gain in BC patients in chemotherapy [22–25], in our study, a mean weight decrease was observed, possibly due to reduced caloric intake and/or increased physical activity after starting chemotherapy. However, we found no association of weight change with total calorie intake, but an increasing risk of weight gain for worsening MD adherence, suggesting that qualitative changes in diet were at least as important as reduced calorie intake for preventing weight gain. Indeed, we observed substantial dietary changes in BC patients in the study period: a reduced consumption of pasta or rice, bread, potatoes, breadsticks or crackers, meat, fat and lean salami, cheese, milk, yogurt, added sugar, icecreams and sweet snacks, soft drinks and fruit juice, alcoholic beverages and butter, and an increased intake of fruit, vegetables, legumes, and fish. The mean total energy intake decreased with a proportional reduction in intake of proteins, carbohydrates, and fats. Overall, the dietary habits of BC patients modified towards a higher adherence to the MD pattern, as confirmed by the mean 0.6 increase in the MDS 0–9 scale [19]. Of note, only 18.5% of women with MDS improvement had weight gain, compared to 44.4% of those with MDS worsening (see Table 4 and supplementary Table 4). These dietary changes were also in agreement with the recommendations of international Agencies and Scientific Associations for healthy diet in cancer survivors [2, 26] and are consistent with the dietary patterns found to be associated with better survival in BC patients [27].

In our study, we found a higher proportion of BC patients who increased or maintained their physical activity than those who reduced it, contrary to studies showing a decrease of physical activity during and after chemotherapy [28]. We found that the risk of weight gain was higher in women who reduced physical activity after BC diagnosis than those who did not, in agreement with other studies showing that physical activity can play an important role in preventing weight gain and improving BC prognosis [29]. The role of adjuvant hormone therapy in weight gain of BC patients, with inconsistent evidence from studies carried out so far, mainly due to methodological issues concerning study design, type of therapy, weight measures, and time frames [8]. The type of adjuvant endocrine treatment may be substantial, with aromatase inhibitors found less likely to determine weight gain than tamoxifen in some studies [8]. In our study, we could not analyze the two therapeutic regimens separately, due to the small number of women who had taken tamoxifen. However, when we restricted the analysis to women who were treated with aromatase inhibitors, we found no substantial difference compared to the whole sample. This study has some strengths, including the prospective design and the collection of clinical, dietary, and physical activity data using structured questionnaires. However, it has some limits, too. Our study was interventional by design, but no control group could be included, since we had no data on the lifestyle of women who did not accept to fill in the proposed questionnaires. A second limit is the lack of measures of body composition in BC patients before and after chemotherapy. Finally, about one quarter of BC patients enrolled in the study did not meet the follow-up the last assessment, preventing us from making an intention-to-treat analysis of the data. However, we found that the non-compliant BC patients were similar to those who had the last assessment as regards the baseline characteristics, and therefore, we are confident that no substantial selection bias occurred, although an overestimate of the beneficial of the intervention restricting the analysis to women with complete follow-up cannot be excluded. Finally, we observed a positive result of the intervention at 18 months after the start, but a longer observation is needed for evaluating persistence of the effect. Really, this is a limit of the weight loss intervention trials carried out so far in BC survivors, with a research need for long-term follow-up to assess the persistence of weight changes [30].

In conclusion, this study showed that monitoring BC patients’ habits before and after chemotherapy and providing simple suggestions as regards the Mediterranean diet principles and regular physical activity according to international guidelines is effective for preventing weight gain in normal weight women and for favoring weight loss in overweight and obese women. All BC patients were offered to follow our lifestyle program after chemotherapy freely, and about 80% of them accepted to participate, 20% of which, however, left the program during the follow-up. Increasing the proportion of BC women who regularly follow a lifestyle intervention seems a real challenge in a public health perspective at present.

### Supplementary Information

Below is the link to the electronic supplementary material.

Supplementary file1 (DOCX 38 kb)

## Data Availability

The datasets generated during and/or analyzed during the current study are available from Rebecca Pedersini and Sara Bosio on reasonable request.
