# Supplementary material for: Is weight gain preventable in women with early breast cancer undergoing chemotherapy? A real-world study on dietary pattern, physical activity, and body weight before and after chemotherapy
Source: Breast Cancer Res Treat. 2023 Sep 11;202(3):461–71. doi: 10.1007/s10549-023-07095-8 (PMC10564810; doi:10.1007/s10549-023-07095-8)
Supplement: Supplementary file 1 — Supplementary file1 (DOCX 38 kb) [file 10549_2023_7095_MOESM1_ESM.docx]

Supplementary Table 1. Baseline clinical characteristics of 46 patients with breast cancer enrolled in the study who did not undergo the final assessment, 12 months after the end of chemotherapy

| **Characteristics** | **Number (%)** |
| --- | --- |
| Age, years: mean ± SD (range) | 52 ±12 (26-75) |
| Menopausal status  Premenopausal  Postmenopausal | 19 (41)  27 (59) |
| Pathological Tumour stage  0  1  ≥2 | 3 (7)  28 (62)  14 (31) |
| Pathological Nodal stage  0  ≥1 | 23 (52)  21 (48) |
| Histological type  No Special Type  Others | 43 (93)  3(7) |
| Estrogen receptor  Positive  Negative | 31 (67)  15 (33) |
| Progesterone receptor  Positive  Negative | 22 (48)  24 (52) |
| Grading  G1 or G2  G3 | 3 (4)  43 (96) |
| Ki-67 labelling index  <20%  ≥20% | 6 (13)  40 (87) |
| HER2  Positive  Negative | 33 (72)  13 (28) |
| Chemotherapy  Adjuvant  Neo-adjuvant | 39 (85)  7 (15) |
| Chemotherapy regimen  Anthracyclines  Taxanes  Sequential  Others | 10 (22)  1 (2)  6 (13 29 (63) |
| Hormone therapy  None  yes | 17 (37)  29 (63) |
| Surgery Treatment  Breast-conserving  Mastectomy | 31 (699  14 (31) |

G1: well-differentiated tumour; G2 moderately differentiated tumour; G3: undifferentiated tumour; HER2: Human epidermal growth factor receptor 2; LHRH: Luteinizing Hormone-Releasing Hormone.

Supplementary Table 2. Food, beverages, and condiments consumption at baseline in 46 patients with breast cancer who did not undergo the final assessment. All measures are expressed as means of grams for food and ml for beverages per week, with the corresponding 95% confidence interval (CI) in brackets.

| Food, Beverage or Condiment | Mean (IC95%) |
| --- | --- |
| Fruit | 2087 (1758;2416) |
| Vegetables | 1765 (1551;1980) |
| Pasta or rice | 424 (361;487) |
| Bread | 493 (399;589) |
| Potatoes | 254 (191;317) |
| Breadsticks or crackers | 85 (48;123) |
| White meat | 253 (199;307) |
| Red meat | 141 (102;179) |
| Fish | 223 (184;262) |
| Lean salami | 92 (67;117) |
| Fat salami | 29 (18;39) |
| Eggs | 149 (103;196) |
| Fresh cheese | 203 (143;262) |
| Aged cheese | 117 (78;156) |
| Legumes | 182 (126;238) |
| Milk | 470 (275;665) |
| Yogurt | 279 (171;386) |
| Biscuits | 127 (85;168) |
| Ice-creams | 83 (44;122) |
| Sweet snacks | 182 (116;248) |

Supplementary Table 3. Food, beverages, and condiments consumption, and energy intake (kcal/day) and MDS score one week before chemotherapy (T0) and 12 months after ending chemotherapy (T6) , in 113 patients treated and 56 patients not treated with hormone therapy. Food consumptions are expressed as means of grams or ml per week, with corresponding 95% CI in brackets.

| Characteristic | **T0** | | | | **T6** | | | |
| --- | --- | --- | --- | --- | --- | --- | --- | --- |
|  | **Hormonotherapy** | |  |  | **Hormonotherapy** | |  |  |
|  | **no** | **yes** | **Δ (no-yes)** | **p*** | **no** | **yes** | **Δ (no-yes)** | **p*** |
| Fruit | 1732 (1426;2037) | 1912 (1682;2141) | -180 (568;209) | 0.336 | 2178 (1903;2452) | 2180 (2004;2356) | -2.63 (-316;311) | 0.865 |
| Vegetables | 1526 (1323;1729) | 1604 (1462;1747) | -78 (-324;168) | 0.550 | 1804 (1668;1939) | 1900 (1796;2004) | -96 (-270;78) | 0.201 |
| Pasta or rice | 393 (338;448) | 393 (355;432) | 0 (-67;66) | 0.960 | 378 (331;425) | 325 (292;359) | 52 (-5;110) | 0.06 |
| Bread | 392 (31;473) | 448 (390;506) | -55 (-155;44) | 0.272 | 285 (230;341) | 302 (258;345) | -16.5 (-89;56) | 0.793 |
| Potatoes | 258 (195;321) | 274 (232;316) | -16 (-90;57) | 0.495 | 220 (174;266) | 203 (171;235) | 17 (-39;72) | 0.527 |
| Breadsticks or crackers | 108 (71;146) | 100 (76;125) | 8 (-35;51) | 0.895 | 28 (8;48) | 31 (19;44) | -3.8 (-26;18) | 0.342 |
| White meat | 271 (221;322) | 227 (199;255) | 44 (-8;97) | 0.179 | 226 (194;258) | 197 (177;218) | 29 (-8;65) | 0.156 |
| Red meat | 135 (105;166) | 135 (113;158) | 0 (-38;39) | 0.897 | 81 (58;104) | 74 (62;86) | 7 (-15;30) | 0.903 |
| Fish | 260 (207;313) | 277 (240;314) | -17 (-80;47) | 0.407 | 356 (295;417) | 364 (329;399) | -8 (-73;57) | 0.645 |
| Lean salami | 99 (74;124) | 99 (82;117) | 0 (-30;30) | 0.976 | 33 (21;43) | 38 (28;48) | -5 (-22;11) | 0.782 |
| Fat salami | 30 (19;41) | 29 (19;38) | 1 (-14;16) | 0.548 | 6 (2;10) | 7 (4;10) | -1 (-7;4) | 0.671 |
| Eggs | 129 (97;160) | 113 (100;126) | 16 (-13;44) | 0.799 | 133 (107;158) | 108 (97;119) | 25 (1;48) | 0.195 |
| Fresh cheese | 232 (179;285) | 184 (151;217) | 48 (-12;107) | 0.054 | 155 (121;189) | 178 (152;204) | -23 (-66;20) | 0.267 |
| Aged cheese | 129 (94;165) | 121 (100;142) | 8 (-30;47) | 0.863 | 62 (44;80) | 60 (48;71) | 2 (-18;22) | 0.842 |
| Legumes | 229 (164;294) | 193 (158;228) | 36 (-31;104) | 0.548 | 309 (251;367) | 327 (289;366) | -18 (-86;49) | 0.536 |
| Milk | 471 (306;637) | 437 (318;556) | 34 (-169;237) | 0.508 | 253 (132;374) | 222 (133;311) | 31 (-120;183) | 0.579 |
| Yogurt | 365 (251;479) | 321 (203;439) | 44 (-141;229) | 0.099 | 69 (21;118) | 131 (78;183) | -61 (-143;19) | 0.280 |
| Added sugar | 102 (70;135) | 62 (48;75) | 40 (11;70) | 0.038 | 29 (19-40) | 23 (13;34) | 6 (-10;23) | 0.029 |
| Biscuits | 168 (131;205) | 119 (95;143) | 49 (6.6;91) | 0.017 | 105 (78;133) | 115 (92;138) | -10 (-48;28) | 0.886 |
| Ice-creams | 110 (68;151) | 73 (49;96) | 37 (-7;61) | 0.061 | 36 (12;59) | 30 (14;46) | 6 (-22;33) | 0.429 |
| Sweet snacks | 216 (126;306) | 157 (115;199) | 59 (-27;145) | 0.113 | 95 (51;139) | 68 (50;86) | 27 (-13;67) | 0.986 |
| Soft drinks | 299 (149;447) | 240 (130;349) | 59 (-128;245) | 0.064 | 34 (6;62) | 68 (8;129) | -34 (-122;53) | 0.859 |
| Fruit juices | 266 (148;385) | 237 (159;315) | 29 (-108;167) | 0.869 | 116 (29;202) | 75 (33;118) | 40 (-45;125) | 0.655 |
| Wine | 261 (137;386) | 326 (229;423) | -65 (-227;97) | 0.316 | 152 (70;235) | 208 (134;282) | -56 (-175;64) | 0.407 |
| Beer | 121 (39;203) | 122 (69;174) | -1 (-95;92) | 0.454 | 71 (32;109) | 61 (36;85) | 10 (-34;54) | 0.787 |
| Schnapps | 5.2 (-0.5-;11) | 2.6 (-0.06;5.19) | 2.6 (-2.8-;8) | 0.470 | 0.18 (-0.18;0.53) | 0.70 (-0.5;1.9) | -0.5 (-2.3;1.24) | 1.00 |
| Olive Oil | 257 (235;280) | 244 (229;259) | 13 (-13;40) | 0.265 | 235 (217;253) | 251 (237;263) | -16 (-38;6.7) | 0.225 |
| Butter | 27 (15;39) | 15 (12;18) | 12 (3;21) | 0.291 | 12 (4;20) | 7 (5;10) | 5 (-2;12) | 0.420 |
| Total energy (kcal/day) | 1847 (1694;2001) | 1707 (1627;1789) | -141 (-296;15) | 0.19 | 1405 (1332;1477) | 1407 (1353;1460) | 2 (-89;92) | 0.76 |
| MDS score (0-9) | 3.9 (3.4;4.3) | 4.4 (4.2;4.7) | 0.6 (0.1;1.1) | 0.043 | 4.7 (4.3;5.0) | 4.9 (4.7;5.2) | 0.3 (-0.2;0.7) | 0.24 |

*Wilcoxon signed-rank test

Supplementary Table 4. Characteristics of the patients according to weight gain vs stable weight or weight gain.

| **Characteristic** | **Stable weight or weight loss**  **N (%)** | **Weight gain**  **N (%)** | **Total**  **N (%)** | **P*** |
| --- | --- | --- | --- | --- |
| Total patients | 137 (81.1) | 32 (18.9) | 169 (100) |  |
| Age  < 49  49-58  >59 | 34 (67)  51 (86)  52 (88) | 17 (34))  8 (14)  7 (12) | 51 (100)  59 (100)  59 (100) | 0.007 |
| Basal BMI  Normal weight/underweight  Overweight  Obesity | 73 (72)  39 (95)  25 (96) | 29 (28)  2 (5)  1 (4) | 102 (100)  41 (100)  26 (100) | 0.001 |
| Menopausal status  No  Yes | 52 (76)  85 (84) | 16 (24)  16 (16) | 68 (100)  101 (100) | 0.211 |
| Pathological Tumour stage  0  1  ≥2 | 15 (78)  68 (83)  54 (79) | 4 (21)  14 (17)  14 (21) | 19 (100)  82 (100)  68 (100) | 0.835 |
| Pathological Nodal stage  0  ≥1 | 79 (83)  58 (78) | 16 (17)  16 (22) | 95 (100)  74 (100) | 0.431 |
| Histologic type  No Special Type  Lobular  Others | 124 (81)  12 (80)  1 (100) | 29 (19)  3 (20)  0 (0) | 153 (100)  15 (100)  1 (100) | 0.885 |
| Estrogen receptor  Negative  Positive | 44 (86)  93 (79) | 7 (14)  25 (21) | 51 (100)  118 (100) | 0.256 |
| Progesterone receptor  Negative  Positive | 63 (90)  74 (75) | 7 (10)  25 (25) | 70 (100)  99 (100) | 0.013 |
| Grading  G1 or G2  G3 | 15 (75)  120 (82) | 5 (25)  27 (18) | 20 (100)  147 (100) | 0.480 |
| Ki-67 labelling index  <20%  ≥20% | 23 (74)  114 (83) | 8 (26)  24 (17) | 31 (100)  138 (100) | 0.280 |
| HER2  Negative  Positive | 79 (77)  58 (87) | 23 (23)  9 (13) | 102 (100)  67 (100) | 0.139 |
| Chemotherapy  Adjuvant  Neo-adjuvant | 105 (81)  32 (82) | 25 (19)  7 (18) | 130 (100)  39 (100) | 0.858 |
| Chemotherapy regimen  Taxane based  Not taxane based | 108 (82)  29 (76) | 23 (18)  9 (24) | 131 (100)  38 (100) | 0.396 |
| Hormone therapy  No  Yes | 50 (89)  87 (77) | 6 (11)  26 (23) | 56 (100)  113 (100) | 0.05 |
| Type of surgery  Conservative  mastectomy | 76 (84)  61 (78) | 15 (16)  17 (22) | 91 (100)  78 (100) | 0.380 |
| Kcal T0-T6  Decrease  Stable  Increase | 88 (80)  30 (83)  19 (83) | 22 (20)  6 (17)  4 (17) | 110 (100)  36 (100)  23 (100) | 0.888 |
| MDS T0-T6  Worsening  Stable  Improvement | 10 (56)  83 (86)  44 (81) | 8 (44)  14 (14)  10 (19) | 18 (100)  97 (100)  54 (100) | 0.012 |
| Physical activity frequency T0-T6  Increase  Stable  Decrease | 61 (86)  60 (83)  16 (62) | 10 (38)  12 (17)  10 (38) | 26 (100)  72 (100)  71 (100) | 0.020 |
